# Hyperbaric Oxygen Preconditioning Induces Tolerance against Oxidative Injury and Oxygen-Glucose Deprivation by Up-Regulating Heat Shock Protein 32 in Rat Spinal Neurons

**DOI:** 10.1371/journal.pone.0085967

**Published:** 2014-01-17

**Authors:** Guoyang Huang, Jiajun Xu, Li Xu, Shifeng Wang, Runping Li, Kan Liu, Juan Zheng, Zhiyu Cai, Kun Zhang, Yuandeng Luo, Weigang Xu

**Affiliations:** 1 Department of Diving and Hyperbaric Medicine, Faculty of Naval Medicine, Second Military Medical University, Shanghai, People's Republic of China; 2 Naval Medical Institute, Shanghai, People's Republic of China; Massachusetts General Hospital/Harvard Medical School, United States of America

## Abstract

**Objective:**

Hyperbaric oxygen (HBO) preconditioning (HBO-PC) has been testified to have protective effects on spinal cord injury (SCI). However, the mechanisms remain enigmatic. The present study aimed to explore the effects of HBO-PC on primary rat spinal neurons against oxidative injury and oxygen-glucose deprivation (OGD) and the relationship with heat shock proteins (HSPs).

**Methods:**

Primary rat spinal neurons after 7 days of culture were used in this study. HSPs were detected in rat spinal neurons following a single exposure to HBO at different time points by Western blot. Using lactate dehydrogenase release assay and cell counting kit-8 assay, the injuries induced by hydrogen peroxide (H2O2) insult or OGD were determined and compared among neurons treated with HBO-PC with or without HSP inhibitors.

**Results:**

The results of Western blot showed that HSP27, HSP70 and HSP90 have a slight but not significant increase in primary neurons following HBO exposure. However, HSP32 expression significantly increased and reached highest at 12 h following HBO exposure. HBO-PC significantly increased the cell viability and decreased the medium lactate dehydrogenase content in cultures treated with H2O2 or OGD. Pretreatment with zinc protoporphyrin IX, a specific inhibitor of HSP32, significantly blocked the protective effects of HBO-PC.

**Conclusions:**

These results suggest that HBO-PC could protect rat spinal neurons in vitro against oxidative injury and OGD mostly by up-regulating of HSP32 expression.

## Introduction

Spinal cord injury (SCI) is a serious health problem, which may result from surgical operations on the spinal column or thoracoabdominal aorta, or decompression sickness (DCS) associated with sport or commercial diving. The reported rate of paraplegia or paraparesis after thoracoabdominal aortic aneurysms surgery varies from 3.8% to 16.7% [1], [2] and the incidence of injured divers with incomplete recovery from neurological DCS has been reported between 20% and 30% [3]. Under those high risk conditions, effective preventive measures are in desperate need to reduce the incidence of SCI.

Hyperbaric oxygen (HBO) therapy is the way that a person breathes pure oxygen under a pressure greater than 1 atmosphere absolute in a specially designed chamber [4]. It is widely used in the management of various diseases and conditions including DCS, carbon monoxide (CO) poisoning, gas embolism and neurologic diseases [5]. As an effective therapy for SCI, HBO has been reported to accelerate neurologic recovery by reversing hypoxia, ameliorating mitochondrial dysfunction, arresting the spread of hemorrhage, reducing edema, up-regulating the activity of antioxidant enzymes and decreasing the production of inflammatory factors 6–8. In addition, recent studies have found that preconditioning with HBO could be an effective preventive measure to alleviate SCI. In 2002, HBO preconditioning (HBO-PC) was first applied in experimental SCI with beneficial effects [9]. Later studies demonstrated that HBO-PC exerted its neuroprotective effects by up-regulating the activity of catalase and superoxide dismutase, promoting axonal growth and suppressing mitochondrial apoptosis pathway [10]–[12]. Our previous work found that HBO-PC alleviated SCI by up-regulating of heat shock protein (HSP) 70 expression in a DCS rat model [13]. However, there is still short of evidence about the direct effects of HBO-PC on spinal neurons in vitro and the mechanisms.

HSPs normally perform as molecular chaperones and are called protein guardians because they play a crucial role in repairing partially damaged proteins [14]. In the setting of SCI, HSPs induction has been shown to be beneficial, which can alleviate SCI through its anti-inflammatory and anti-apoptotic actions [15], [16].

The aim of the present study was to observe the protective effects of HBO-PC on primary rat spinal neurons, and to explore the possible roles of HSPs.

## Materials and Methods

The experimental procedures were carried out in accordance with the National Institutes of Health Guidelines on the Use of Laboratory Animals and approved by the Institutional Animal Care and Use Committee of the Second Military Medical University.

### Cell culture

Primary culture of rat spinal neurons was done using the method described by Jiang et al. with minor modifications [17]. Spinal neurons were obtained from embryonic day 14–15 Sprague-Dawley rats. The spinal cords were rapidly dissected from embryo and cut into 1 mm^3^ slices in a 35 mm diameter dish at 4°C. The slices were then digested in pre-warmed 0.05% trypsin (Invitrogen, USA) at 37°C for 18 min in a conical flask agitated by hand every 5 min. After digestion, the supernatant was removed and the remained trypsin was inactivated with Dulbecco's modified Eagle's medium (DMEM; Invitrogen) supplemented with 20% heat-inactivated fetal bovine serum (Invitrogen) at room temperature (RT). Afterwards, tissues were centrifuged for 5 min at 1,500 rpm. The supernatant was removed and the tissues were resuspended in DMEM containing 10% heat-inactivated fetal bovine serum, 10% heat-inactivated horse serum (Invitrogen) and triturated 15–20 times with a fire-polished Pasteur pipette. After the slices were let to settle, single cell suspensions were collected, counted with a hemocytometer, and diluted to a density of 1×10^6^ cells/ml. The cells were plated onto poly-L-lysine- (molecular weight, 30,000–70,000; Sigma, USA) coated culture plates at a density of 1.2×10^6^ cells/well on 6-well plates (for western blot) or 3×10^5^ cells/well on 12-well plates (for lactate dehydrogenase assays; LDH) or 1×10^5^ cells/well on coverslips (for immunocytochemistry) or 0.5×10^5^ cells/well on 96-well plates (for cell counting kit-8 assays; CCK-8), then the cultures were kept at 37°C in a 5% CO_2_ humidified incubator. Four hours later, the medium was replaced with serum-free neurobasal medium (Invitrogen) supplemented with 2% B27 supplement (Invitrogen), 100 U/ml penicillin, 100 µg/ml streptomycin, and 0.5 mM glutamine (Invitrogen). 25 µM glutamate (Invitrogen) was added during the first day in vitro. On the second day, 5 µM cytosine-β-D-arabinofuranoside (Sigma, USA) was added into the medium for 24 h to inhibit non-neuronal cell division. After that, half volume of culture media was replaced with fresh media every 3 days. The cultures consisted of more than 90% neurons as identified by immunocytochemistry staining and those cultures were utilized for the experiments described below at 7 days in vitro.

### Identification of cultured cells

Rat spinal neurons were identified by immunocytochemistry staining. Briefly, at 7 d in vitro, cultures on PLL-coated coverslips were fixed with 4% paraformaldehyde (PFA) for 30 min, and rinsed three times with 0.01 M phosphate-buffered saline (PBS) and blocked with 5% BSA for 20 min at RT. The monoclonal rabbit anti-rat primary antibody against β-tubulin III (Sigma) was added to the cell slides and co-cultured with the cells in a humidified chamber overnight at 4°C. After three washes with PBS, cultures were incubated for 1 h at 37°C with the HRP-conjugated goat anti-rabbit antibody (Sigma). Then cultures were stained with peroxidase substrate diaminobenzidine. 4′, 6-diamidino-2-phenylindole (DAPI; Sigma) was used to stain nuclei of all cells, including neurons and glial cells. The purity of cultured spinal neuron was expressed as the percentage of β-tubulin III positive cells relative to the total number of DAPI labeled nuclei.

### Hyperbaric oxygen preconditioning

HBO exposure was performed in a temperature and humidity controlled hyperbaric incubator (OxyCure 3000, OxyHeal® Health Group, USA). The pressure-duration was 280 kPa-60 min, which is frequently used in animal and cell study. The compression and decompression were both carried out within 5 min. The chamber was flushed and compressed with pure oxygen containing 1.79% CO_2_. Thus, at pressure of 280 kPa, the partial pressure of CO_2_ reached 5 kPa to maintain a physiological pH of the culture. All the pressures described in this text are absolute pressures.

### Determination of heat shock protein

HSP27, 32, 70 and 90 were determined at 0, 6, 12, 18, 24, and 30 h following HBO exposure by Western blot and verified by immunofluorescence staining.

#### Western blot

The neurons were harvested and lysed in Radio-Immunoprecipitation Assay Lysis Buffer (Sigma). Protein samples were electrophoresed on 8% SDS-polyacrylamide gels, and transferred onto a polyvinyldifluoridine membrane (Millipore, USA) and detected with rabbit monoclonal primary antibodies directed against rat HSP27 (Cell Signaling Technology, USA), HSP32 (Abcam, USA), HSP70 (GeneTex, USA), HSP90 (Cell Signaling Technology) or β-actin (Abcam). Proteins were visualized by using HRP-conjugated goat anti-rat IgG (Abcam) and the intensity of each band was measured by using Kodak Digital Science 1D Image Analysis System (Eastman Kodak, USA).

#### Immunofluorescence staining

The results of western blot showed that only HSP32 was significantly expressed (see in Results). To verify the expression, the cultures on PLL-coated coverslips were fixed with 4% PFA for 30 min, blocked with 5% BSA for 20 min at RT and incubated with monoclonal rabbit anti-rat primary antibody against HSP32 (Abcam) overnight at 4°C and then with the FITC-conjugated goat anti-rabbit IgG (Sigma) for 1 h at 37°C, and mounted with Gel/Mount aqueous mounting media containing DAPI. Then cultures were observed under a fluorescence microscope.

### Determination of the effects of HBO-PC on neurons against injuries

#### Groups and treatments

Neuron injuries were induced at 12 h after the HBO-PC using hydrogen peroxide (H_2_O_2_) or oxygen-glucose deprivation (OGD). Cultures were randomly divided into nine groups (n = 4, each with 6 parallel wells): (1) Air, (2) HBO, (3) Air +H_2_O_2_, (4) Air +OGD, (5) HBO+H_2_O_2_, (6) HBO+OGD, (7) HBO+H_2_O_2_+Zn-pp, (8) HBO+OGD+Zn-pp, (9) Air +Zn-pp. For H_2_O_2_ mediated oxidative injury, H_2_O_2_ (Sigma) was diluted into the culture medium at a final concentration of 200 µM and maintained for 4 h at 37°C. For OGD, the cells were cultured with sugar-free Earle's solution (in mmol/L: 121.7 NaCl, 0.8 MgSO_4_, 20.7 NaHCO_3_, 5.5 KHCO_3_, 1 NaH_2_PO_4_, 1.8 CaCl_2_, 0.01 glycine, and 10 HEPES, pH 7.4) in an anaerobic chamber (Thermo Forma Scientific, USA) that was filled with an anoxic gas mixture (5% CO_2_ and 95% N_2_) at 37°C for 2 h and for another 24 h under normal conditions. Control cells were incubated in the same solution with glucose (4.5 g/L) under normal conditions. Znic protoporphrin IX (Zn-pp; Sigma), a specific inhibitor of HSP32, was dissolved in complete dimethyl sulfoxide (DMSO, the final concentration in the culture medium was 0.15%), and added into the cultures immediately before HBO treatment at a final concentration of 3 µM. Group 8 and 9 were adopted to show whether HBO or Zn-pp alone has effects on the determined parameters.

#### Injury parameter determination

To determine whether the protocol of HBO-PC can protect rat spinal neurons from H_2_O_2_-induced oxidative injury or OGD, we detected the cell viability and the medium lactate dehydrogenase (LDH) content after those injuries using cell counting kit-8 (CCK-8) assay (Dojindo Laboratories, Japan) and LDH release assay (Nanjing Jiancheng Bioengineering Institute, China), respectively. Medium LDH content was measured 24 h after injuries following the manufacture's instruction. After injuries, the medium was replaced with normal medium and CCK-8 solution was added to the cell culture medium at a final concentration of 10 µl/100 µl, and incubated for an additional 3.5 h under normal conditions, the absorbance at 450 nm were measured.

### Statistical analysis

All data are presented as mean ± standard deviation (SD). Statistical analysis was made using one-way ANOVA and was performed with SPSS software (version 16.0). A P-value of less than 0.05 was considered to be statistically significant.

## Results

### Identification of spinal neurons

**Figure 1:**
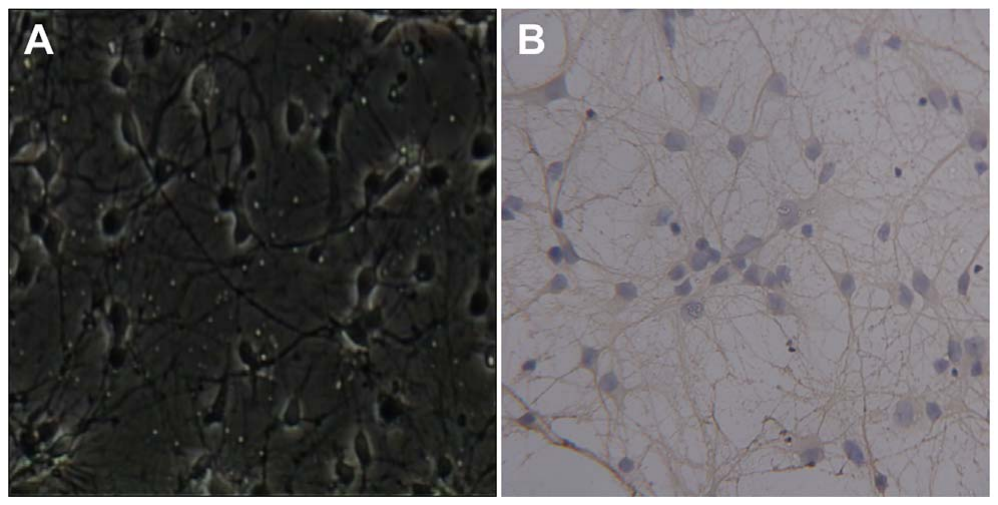
Primary cultured rat spinal neurons. (A) Spinal neurons 7 days in vitro (×200). (B) Immunocytochemistry staining shows that over 90% of the cells counterstained with 4′, 6-diamidino-2-phenylindole (DAPI) were immuno-positive for β-tubulin III, a marker of neurons, which confirms their neuronal identity (×200).

Immunocytochemistry staining of cultures at 7 d in vitro showed that over 90% of the cells counterstained with DAPI were immuno-positive for β-tubulin III, a marker of neurons, which confirmed their neuronal identity (Figure 1).

### HSP expression after HBO exposure

**Figure 2:**
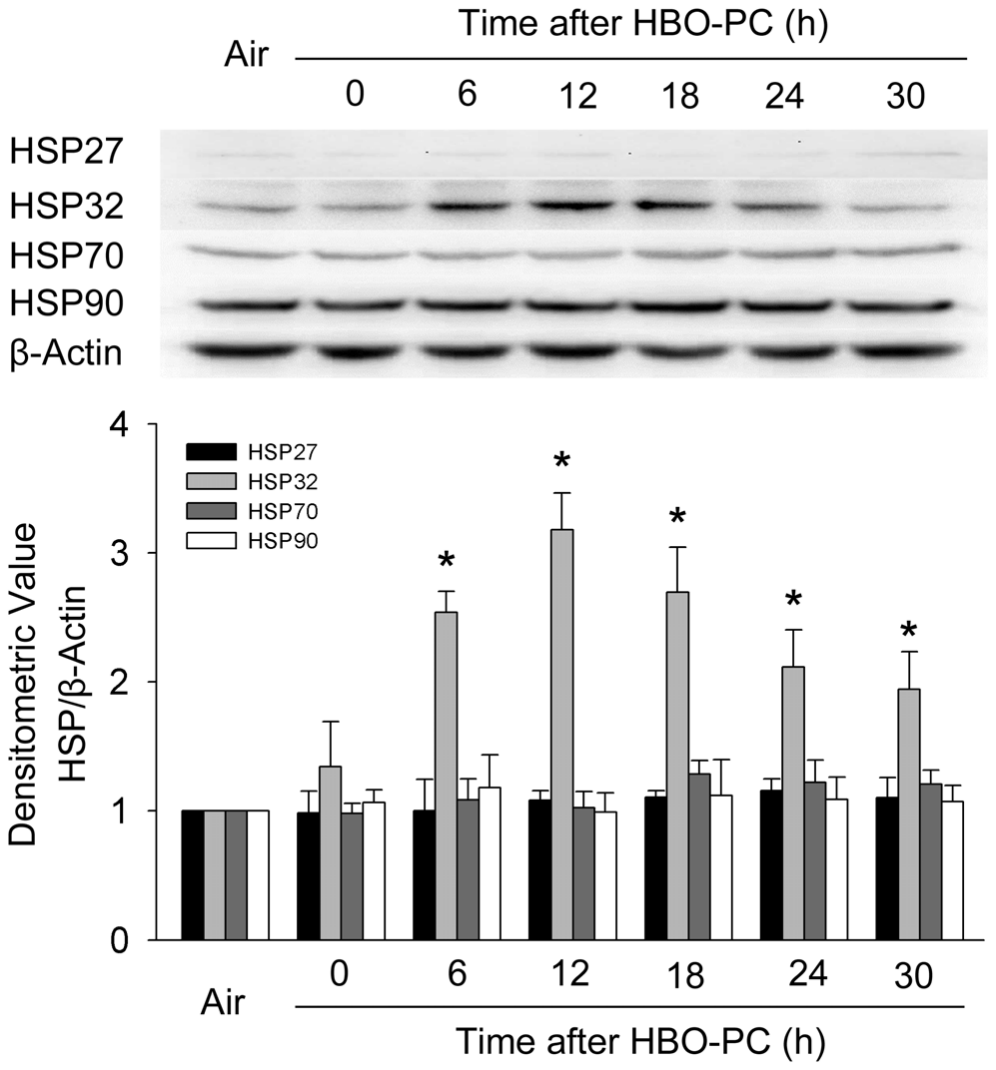
HSP expression in rat spinal neurons at different time points following a single HBO exposure. Western blot shows HSP32 expression increased significantly and reached a peak level at 12-significantly. HBO-PC: hyperbaric oxygen preconditioning, *P<0.01 *vs.* Air group.

**Figure 3:**
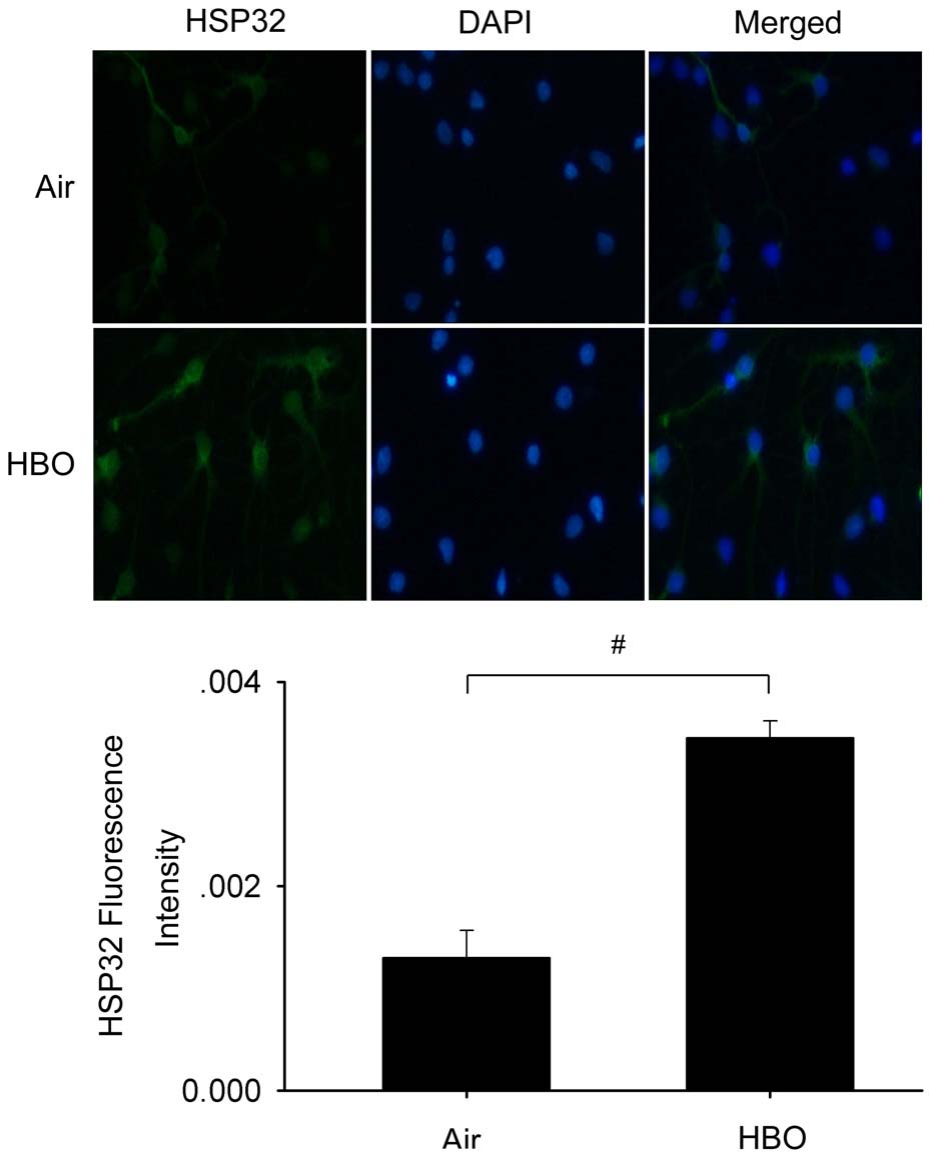
The expression of HSP32 at 12-PC. Immunofluorescence staining verified that HSP32 expression significantly increased. Cell nuclei were labeled with DAPI. # *P*<0.01 *vs.* Air group.

The results of Western blot showed that HSP27, HSP70 and HSP90 had a slight and non-significant increase after HBO exposure, while for HSP32, it increased and reached at a peak level at 12 h after HBO exposure (*P*<0.01; Figure 2). Immunofluorescence staining further verified that the expression of HSP32 significantly increased in neuronal cultures at 12 h after HBO-PC (Figure 3). Therefore, the 12 h time point following HBO exposure was selected to observe the effects of HBO-PC.

### The effects of HBO-PC on spinal neurons against injuries

**Figure 4:**
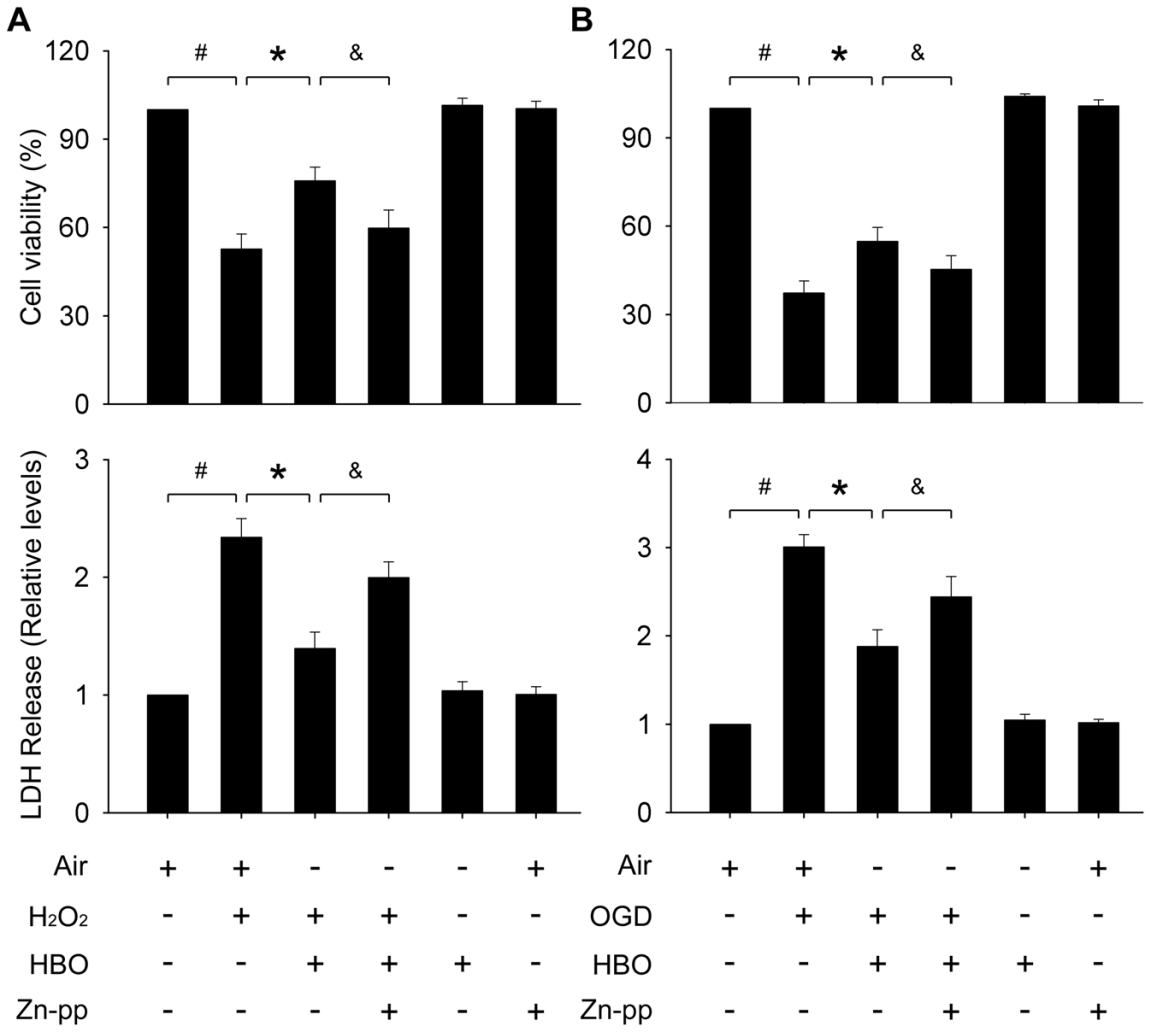
Cell viability and LDH levels in culture medium after oxidative injury or OGD insult. HBO-PC significantly increased the viability of neurons and decreased the medium LDH content. (A) H_2_O_2_ injury, (B) OGD insult. Pretreatment with Zn-pp (3 µM) significantly blocked the protective effects. HBO or Zn-pp has no effects on the parameters under normal conditions. #, *, &: *P*<0.01.

As shown in Figure 4, both H_2_O_2_ insult and OGD significantly decreased the cell viability and increased the medium LDH content compared with the normal control (*P*<0.01). HBO-PC significantly increased the cell viabilities and decreased the medium LDH contents (*P*<0.01). HBO has no effects on the viability or LDH release of the neurons under normal conditions.

### The role of HSP32 in HBO-PC on spinal neurons against injuries

To explore the role of HSP32 in HBO-PC induced neuroprotection, Zn-pp was added into the medium at the final concentration of 3 µM immediately before HBO exposure. Pretreatment with Zn-pp significantly offset the protective effects induced by HBO (*P*<0.01; Figure 4). Zn-pp at 3 µM has no effects on the viability or LDH release of the neurons under normal conditions.

## Discussion

SCI includes primary and secondary injury processes. Primary injury is immediate and irreversible, which results in direct damage to spinal cord tissues. The delayed secondary damage defines a cascade of chemical and physiological events that are initiated by an original insult including ischemia-reperfusion injury (IRI), edema, inflammation, excitotoxicity and oxidative cell damage [18], [19]. Among those events, oxidative stress and IRI are two pivot mechanisms [18]. It has been proved that HBO-PC is effective to increase the ability of spinal cord to counteract oxidative stress and IRI insults in rats and rabbits [10], [12], [13].

In this study, we detected the protective effects of HBO-PC on primary rat spinal neurons against oxidative injury induced by H_2_O_2_ or OGD, a cell IRI model. The results showed that a single episode of HBO exposure 12 h before significantly enhanced the ability of spinal neurons to counteract the oxidative or OGD insults, which is related to HSP32 expression.

As a safe and clinically viable therapy, HBO has been widely used in the management of various diseases and conditions including DCS, carbon monoxide poisoning, gas embolism and neurologic diseases [5]. In addition to elevating the partial pressure of oxygen, HBO causes moderate oxidative stress and further induces the expression of cell protective proteins, which can enhance the cellular tolerance against harmful stimuli [20].

HSP family plays a crucial role in maintaining cell homeostasis and survival against various harmful stimuli [21]. The inducible members including HSP 27, 32, and 70 are associated with cellular protection [22]. HSP90 is constitutive and regulates numerous client proteins to counteract various injuries and in some circumstances is also inducible [23], [24]. HBO has been found to enhance the expression of the above HSPs in liver, heart, brain, kidney and some cell lines [25]–[28]. Our previous work found that HBO-PC significantly induced the expression of HSP70 in rat spinal cord and lung, and contributed to the protection against DCS injuries [13]. A significant increasing of HSP70 expression but not others was also observed in human umbilical vein endothelial cells after a single exposure to HBO (our unpublished data). However, in this study only HSP32 increased significantly and reached a peak level at 12 h following a same profile of HBO exposure. These results indicate that HBO induces different subtype of HSPs in different organs or cells, in vivo or in vitro. The underlying mechanisms deserve further study.

In this study, the protective effects of HBO on rat spinal neurons were significantly blocked by Zn-pp, which can effectively compete for iron protoporphyrin, the natural substrate of HSP32, and inhibit enzyme activity [29]. This and the increased level of HSP32 expression following HBO exposure suggests that the beneficial effects of HBO on rat spinal neurons against oxidative injury and OGD were achieved by up-regulating the expression of HSP32.

HSP32, also known as heme oxygenase-1, is one of the rate-limiting enzymes in heme catabolism, which leads to the generation of biliverdin, ferrous iron, and CO [30]. Recent data indicates that CO can exert anti-inflammatory and anti-apoptotic effects by modulating the MAPK-signaling pathways or activating soluble guanylate cyclase [31]. Ferrous iron released from heme can maintain cells with a low level of iron pools and enhance cell antioxidant capacity by increasing the expression of ferritin and iron ATPase pump [32]. The beneficial roles of biliverdin and bilirubin are to act as physiological antioxidants by efficiently scavenging peroxyl radicals [33]. The exact mechanism by which HSP32 exerts its neuroprotective effects in vitro warrants further studies.

A previous study has reported that HBO-PC protected primary cultured mice spinal neurons against H_2_O_2_-induced oxidative injury via up-regulating the expression of HSP32 [34]. However, in that study, the profile of HBO treatment used was 350 kPa, 98% O_2_ and 2% CO_2_ for 2 h. Except for the higher pressure of O_2_ never used in real practice, the partial pressure of CO_2_ in the hyperbaric mixture reached 7 kPa, which was significant higher than in the sham pretreatment groups and control groups (2 kPa), and is higher than that required to maintain the acid-base equilibrium for the culture medium (5 kPa). Thus, the influence of CO_2_ on the results cannot be ruled out.

In conclusion, this study revealed that a single HBO exposure increased the ability of primary rat spinal neurons to counteract oxidative or OGD injuries mostly by up-regulating the expression of HSP32. Whether a double or multi-exposure further enhances the expression of HSPs and simultaneously the protective effects deserve further study. As a feasible and safe treatment modality, HBO-PC may be a promising way to alleviate the possible SCI in some scheduled operations, such as surgeries on thoracoabdominal aorta and spinal column and diving practice.
